# Trio whole exome sequencing in Chinese childhood-onset lupus reveals novel candidate genes

**DOI:** 10.1002/art.43243

**Published:** 2025-07-21

**Authors:** Jianyang Ma, Yuting Qin, Soon-Min Hong, Thuvaraka Ware, Guojun Hou, Jingjing Tan, Chengmei Xie, Pingjing Zhang, Xiaoqian Wu, Todor Arsov, Lanfang Cao, T Daniel Andrews, Philip Wu, Qian Shen, Huihua Ding, Nan Shen, Carola G. Vinuesa, Yuke He

**Affiliations:** 1 China-Australia Centre for Personalized Immunology (CACPI), Department of Rheumatology, Renji Hospital, Shanghai Jiao Tong University School of Medicine (SJTUSM), Shanghai, China, 200001; 2 Shanghai Institute of Rheumatology, School of Medicine, Renji Hospital, Shanghai Jiao Tong University, Shanghai, China, 200001; 3 Department of Pediatrics, Renji Hospital, Shanghai Jiao Tong University, Shanghai, China, 200001; 4 Faculty of Medical Sciences, University Goce Delcev, Shtip, North Macedonia; 5 Division of Immunology and Infectious Disease, John Curtin School of Medical Research, Australian National University, Canberra, Australian Capital Territory, Australia; 6 The Francis Crick Institute, London, UK

**Keywords:** Systemic lupus erythematosus (SLE), whole exome sequencing (WES), rare variants, de novo variants, type I interferon

## Abstract

**Objectives:**

Systemic lupus erythematosus (SLE) is an autoimmune disease in which rare and common gene variants contribute to pathogenesis. Severe sporadic disease in children is often explained by ‘de novo’ variants that can be uncovered by trio sequencing.

**Methods:**

Whole exome sequencing was performed in 50 Chinese trios with childhood-onset SLE (cSLE). Rare coding variants in SLE-associated genes and all de novo variants were investigated. Gene pathway and expression analysis, and interferon-β luciferase assays were used to predict contribution to disease.

**Results:**

Each proband carried at least one rare variant in an SLE-associated gene with a median of six per child. At least two probands had monogenic disease and one third carried novel or rare variants in genes well accepted to cause monogenic SLE: ACP5, C3, C4A, C4B, DNASE1, IFIH1, NRAS, RNASEH2B, RNASEH2C and SAMHD1. Probands carried a median of one de novo, rare, coding variant. Intriguingly, although only 2 de novo variants occurred in genes previously associated with SLE, 12 of the 50 genes were enriched in the top 20 SLE-related pathways and were highly expressed in ABC/plasma B cells. These genes represent promising candidate lupus genes. Two de novo variants occurring in genes not previously linked to SLE or autoimmunity, DHX8 and ACTR5, enhanced type I IFN signaling.

**Conclusions:**

This study highlights the abundance of lupus-relevant rare gene variants in cSLE, supports frequent contribution of de novo variants to disease, and identifies genes that may constitute novel therapeutic targets of relevance to Chinese patients.

## Introduction

Systemic lupus erythematosus (SLE) is the prototypic systemic autoimmune disease, driven by a combination of genetic and environmental factors. Over the past decades, GWAS have identified hundreds of variants with statistically significant association to SLE (p<1x10^-8^). However, most of these variants are common variants and located in non-coding regions, generally exerting small effects on disease susceptibility^1^. With the advent of next-generation sequencing technologies, many novel and rare variants have been identified in recent years. Some, but not all of these variants are found within genes previously implicated in SLE^2^. Generation of mouse models carrying the orthologous variants has identified novel genes causing monogenic or oligogenic SLE such as *TLR7, UNC93B1, SAT1* and *TNIP1*^3–6^, expanding the list of monogenic lupus causes. Functional validation of variants has also revealed how rare variants in genes like *PACSIN1, SH2B3*, and *P2RY8* likely contribute to SLE susceptibility^7–9^.

Trio testing increases rare disease diagnosis rates, enabling identification of biallelic, homozygous and de novo variants. The latter have been shown to explain two thirds of severe sporadic genetic disease in childhood^10^. Several small studies have investigated the presence of de novo variants in Caucasian cohorts, revealing putative novel causes of monogenic disease. In this study, we set out to investigate the presence, mode of inheritance, and putative contribution of rare gene variants in 50 Chinese probands suffering from childhood-onset SLE (cSLE), which is known to present with more severe phenotype and have a higher genetic risk^11^.

## Materials and Methods

### Human samples collection and sequencing

This study includes 50 unrelated childhood-onset SLE probands from the pediatric department of Shanghai Renji Hospital. All patients fulfilled American College of Rheumatology (ACR) criteria for SLE^12^. The study was approved by the Renji Hospital Ethics Committee of Shanghai Jiaotong University School of Medicine (KY2022 -023-B). Written informed consents were obtained from all participants. Detailed patient characteristics are included in Supplementary Table 1.

Genomic DNA was extracted by using QIAamp DNA Blood kit (QIAGEN) according to the manufacturer’s instructions. WES was performed using illumina HiSeq X platform. Bioinformatic analysis was undertaken by the CACPI pipeline^9^. Deleterious of variants were predicted based on PolyPhen-2, SIFT and CADD. Variants with low minor allele count (<5) were filtered out.

### Gene selection and variant screening

We generated a list of all identified lupus-associated genes including 49 monogenic causes of SLE^13, 14^, and 213 GWAS SLE loci^13, 15^ (Supplementary Table 2). In the case of parent-proband trios, *de novo* origin of variants was established by comparison with their parents’ WES data. Relatedness of probands and parents was confirmed using Peddy^16^. We refer to variants with minor allele frequency [MAF] < 0.005 as “rare variants”. Variants with MAF greater than 0.005 in gnomAD v4.0 and gnomAD v4.0 EAS databases were excluded.

### Gene enrichment analysis

All genes with rare variants were analyzed by MetaScape online analysis tool (https://metascape.org/). The top 20 pathway enrichment plots and gene lists clustered in each pathway were automatically generated by the tool.

### Luciferase reporter assay

HEK293 cells were transfected with IFN-β reporter, renilla reporter, MYD88 plasmid, IRF7 plasmid and indicated plasmids. Cells were lysed 24 hours post-transfection and luciferase activity was detected by using Dual-Glo® Luciferase Assay System (Promega).

### Trio-WES identifies novel candidate genes in cSLE

#### Quantitative real-time RT−PCR

cDNAs were prepared using PrimeScript RT Reagent Kit (Takara) and amplified with the primers shown in Supplemental Table 3. CT values was normalized by GAPDH and relative expression was calculated using the 2−ΔΔCt method.

#### Statistical analysis

For all the experiments, p values were determined by unpaired t test, Mann-Whitney t test, or Chi-Square. P values less than 0.05 were considered significant. Data analysis was performed using Graph Prism 8.0.

## Results

### Characteristics of the childhood-onset SLE cohort

**Figure 1:**
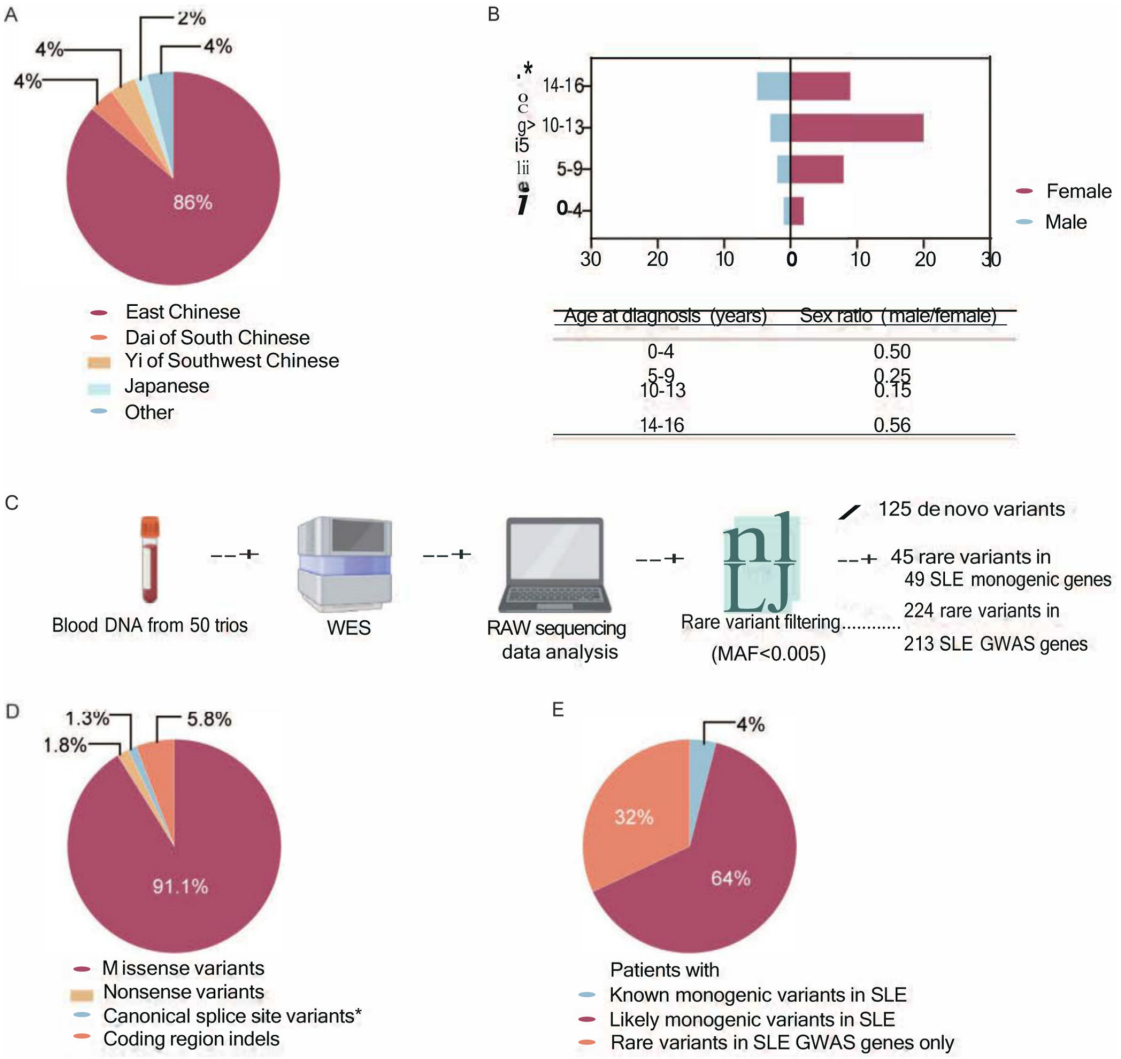
Patient characteristics and the analysis pipeline. (A) Ethnicity of the 50 SLE probands. (B) Age and sex distribution. (C) Schematic of the pipeline for rare variant identification. Rare variants are defined as variants with a minor allele frequency (MAF) of <0.005 (gnomAD EAS v4.0). *De novo* origin of variants was established by comparison with their parents’ sequence data. Putative lupus-associated variants were selected based on filtering rare variants in 49 SLE monogenic genes and 213 GWAS genes. (D) Proportion of variants called by WES. *, splice site variants affecting +1 and +2 nucleotides at the 5′ donor splice site and −1 and −2 residues at the 3′ acceptor splice site. (E) Proportion of patients with different types of variants.

We performed WES of 50 Chinese trios, which included a total of 50 probands and one identical twin sister with childhood-onset SLE. Genetically determined ethnicity revealed that 86% of probands were of Eastern Chinese descent, while Dai of South China and Yi of Southwest China each accounted for 4% (Figure 1A); one child had detectable Japanese ancestry. The mean age at disease onset was 10.8 years, and 78.4% of the probands were female (Table 1). The mean SLEDAI score of 50 probands is 9.96, as determined by the SLEDAI-2K (Table 1).

### Landscape of rare variants identified by WES

Following WES and subsequent data analysis of the 50 trios, we filtered variants by minor allele frequency (MAF) <0.005 according to gnomAD v4.0 EAS. Four types of variants were included: missense variants, nonsense variants, coding region indels, and splice site variants with high likelihood of resulting in alternative splicing. We identified 45 rare variants across 49 genes implicated in monogenic SLE and 224 rare variants across 213 SLE GWAS genes (Figure 1C, Supplementary Table 4). The probands carried a combined 125 de novo rare variants (Figure 1C, Supplementary Table 4). Over 90% of the identified variants were missense variants, and the remainder were nonsense/indel/splice variants (Figure 1D). Pathogenicity assessments revealed that 21.6% of de novo rare variants and 42% of rare variants in SLE-associated genes were predicted to be likely damaging based on PolyPhen-2, SIFT, and CADD scores (Supplementary Figure 1A). Stratification of probands by age at diagnosis showed that the 0–9 years group had a slightly higher number of rare variants in SLE genes compared to the 10−15 years group, though the difference was not statistically significant (Supplementary Figure 1B).

#### Pathogenic variants

**Figure 2:**
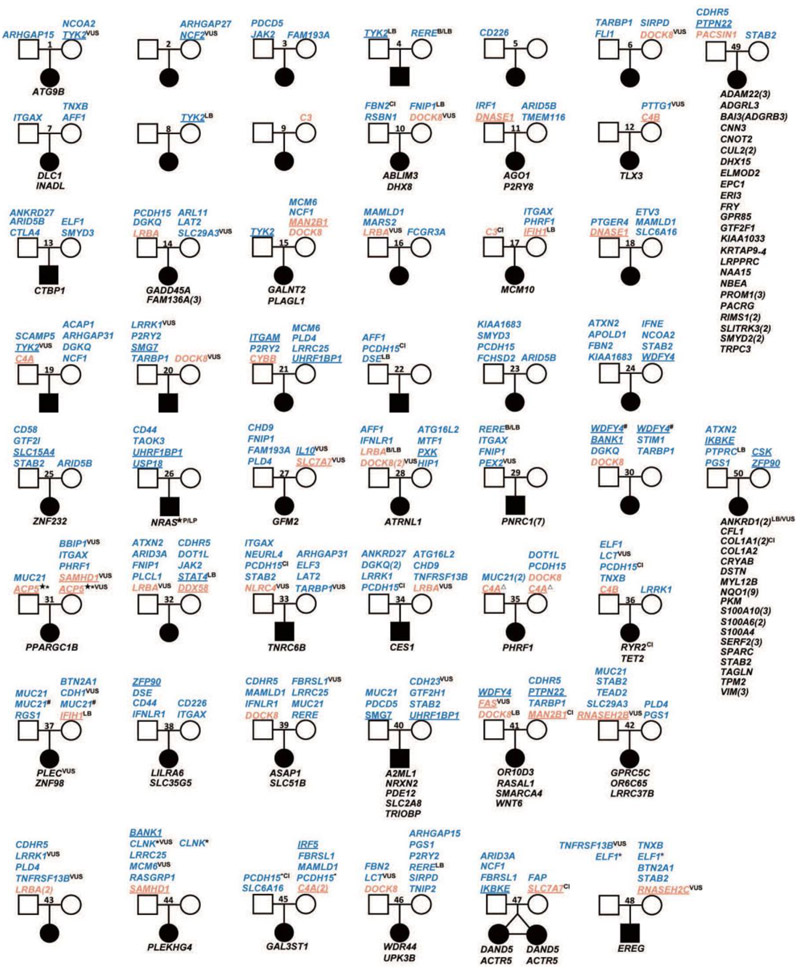
Landscape of rare variants identified by WES in 50 SLE trios. Rare/novel variants in monogenic SLE genes (orange), GWAS (blue) SLE genes and genes with de novo variants (black), carried by each proband were shown in the pedigree. Paternally inherited gene variants (present in the proband) were listed in the upper left, maternally inherited gene variants (present in the proband) were listed in the upper right, and genes with de novo variants were list below each proband. For genes with multiple variant sites, the number of the variants is shown in parentheses. Monogenic SLE genes with known causal variants: ★; Variants inherited paternally or maternally: #; Compound heterozygous: *; Homozygous: △. ClinVar categories: VUS, Variant of uncertain significance; B, Benign; LB, Likely benign; P/LP, Pathogenic/Likely pathogenic; CI, Conflicting interpretations of pathogenicity. SLE genes validated by expression quantitative trait loci (eQTL) analysis are underlined.

Among the 50 lupus trios, we could only confidently identify two probands with monogenic SLE, without further functional validation (Figure 2). Proband 26 carried a de novo G12D variant in *NRAS* that is pathogenic in Noonan syndrome according to ClinVar (Variation ID: 39648); Noonan Sd can present with SLE^17^. This variant has also been found in patients with myeloproliferative disorder^18^ and as a somatic variant in multiple types of cancer such as non-small cell lung carcinoma^19^, multiple myeloma and acute myeloid leukemia^20^. Proband 31 had compound heterozygous variants in *ACP5* - G290V and R46Q - as well as an R24H variant in *SAMHD1* that we have previously reported^21^. ACP5 deficiency has been shown to cause SLE in an autosomal recessive fashion. ACP5 and SAMHD1 variants may act synergistically augment type I IFN production.

We also previously reported the damaging L257F variant in *P2RY8* in proband 11^9^. This is a novel *de novo* variant resulting in a complete loss of function, which leads to a reduced ability to repress AKT activation and migration, along with deregulated ERK activation. This disruption is likely to disrupt B cell tolerance and contribute to the childhood-onset SLE. Given the lack of a mouse *P2RY8* orthologue and in the absence of other kindreds where monogenic disease can be clearly established, it is not possible at this stage to state that P2RY8 loss-of-function causes monogenic SLE.

Whilst proband 35 was homozygous for a novel C4A missense variant (rs28357076) only complete C4 deficiency, which requires four null C4A and C4B alleles carries a 75% incidence of SLE^22^. Although this variant had a MAF = 0.002091 according to gnomAD 4.0 EAS, gnomAD 2.0 recorded a variant frequency is 0.03653 in Eastern Asian individuals. The variant was also predicted to be benign by three algorithms. Thus, it is unlikely this variant causes monogenic disease.

#### Rare variants in putative lupus-causing genes

We next looked at rare variants reported in the literature to cause monogenic SLE or lupus-like disease. Out of the 50 kindreds and excluding the probands described above, an additional 12 probands had rare variants in genes that are well accepted to cause monogenic SLE via either the complement pathway (*C3, C4A and C4B)* or via metabolism of nucleic acids *(DNASE1, IFIH1, RNASEH2B, RNASEH2C* and *SAMHD1)*. All variants were of unknown or uncertain significance except for one *IFIH1* variant in two probands (17 and 37) reported as likely benign in ClinVar. When we included the remaining list of genes suggested to cause SLE in at least one publication, we identified an additional 19 probands. These additional putative mono/oligogenic SLE genes are involved in type I IFN regulation (*DDX58, NLRC4* and *PACSIN1*), disruption of B cell and T cell tolerance (*DOCK8, FAS* and *LRBA*) and metabolic disorders (*CYBB, MAN2B1, SLC7A7*) (Figure 2). Thus, a total of 64% (Figure 1E) of the probands had rare variants of unknown significance in genes with the potential to cause SLE.

Additionally, we identified 224 rare variants in genes associated with SLE by GWAS. Notably, all 50 probands carried rare variants in SLE GWAS genes, with a median of 4.5 per proband (and a total median of 6 variants per proband in SLE-associated genes). In 16 of these probands, rare variants were identified exclusively in SLE GWAS genes, representing 32% of the cohort (Figure 1E).

**Figure 3:**
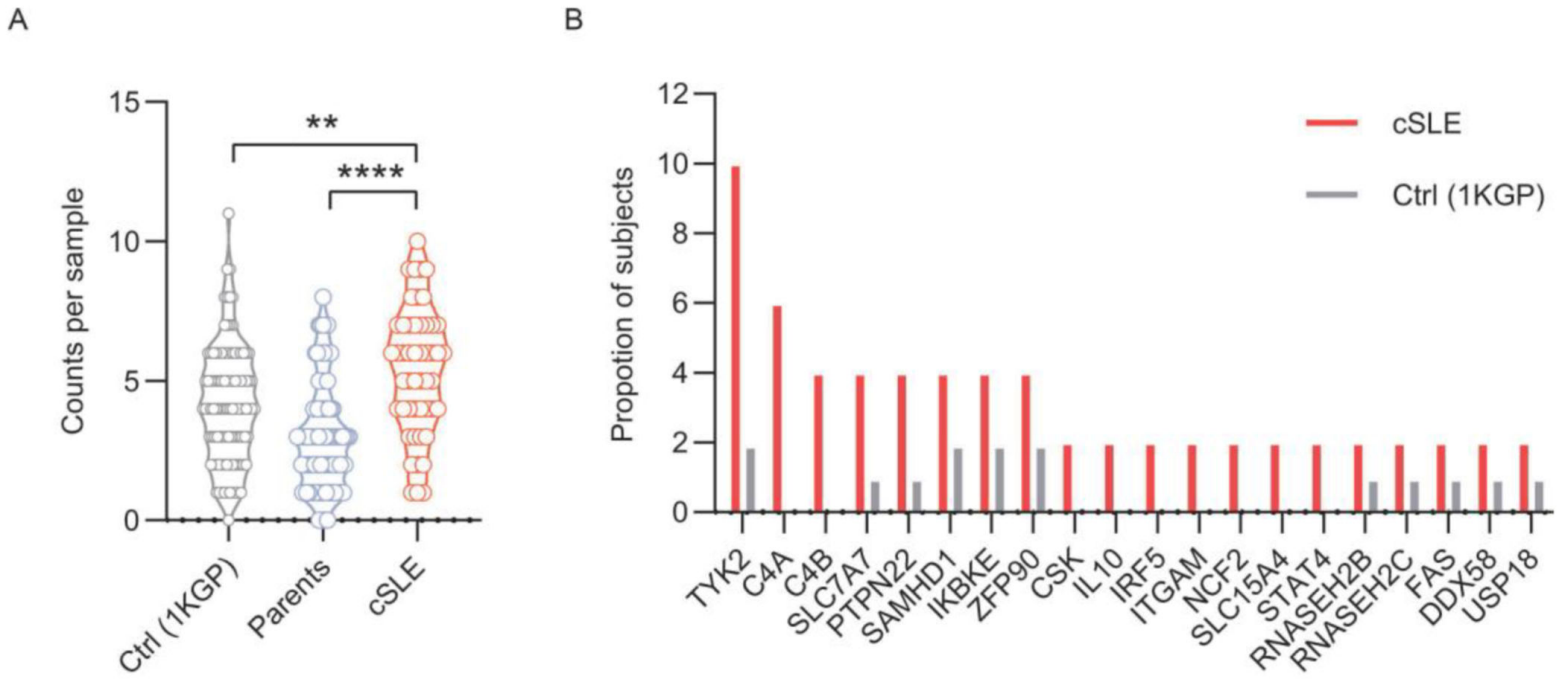
The most frequently mutated genes in 50 SLE trios. (A) Number of rare variants in known SLE genes (monogenic and GWAS SLE genes) per individual in SLE cohort compared with unaffected parents and controls from the 1000 Genomes Project (1KGP). P values determined by Chi-Square test (*P < 0.05, **** P<0.0001). (B) Most frequently mutated genes in SLE cohort, shown as the proportion of SLE patients and controls carrying rare variants in each indicated SLE-associated gene. Data are based on 64 SLE genes validated by expression quantitative trait loci (eQTL) analysis. Genes with variants occurring at≥2 times the prevalence in controls were shown.

We also conducted a comparative analysis using 105 control samples of Han Chinese Southeast Asian ancestry from the 1000 Genomes Project (1KGP) database. This analysis revealed a significant increase in the number of rare variants in known SLE genes within the SLE cohort compared to controls (Figure 3A). These results add to other published evidence that SLE patients carry a higher burden of rare variants in SLE-associated genes^23^. Even though unaffected parents do not constitute an adequate control group for this analysis due to sharing ~50% of their genetic material with probands - this can obscure a disease - specific signal, the parents vs probands comparison remained significant in our cohort (Figure 3A).

A more restricted analysis focused on 64 well-established SLE-associated genes that colocalize with expression quantitative trait loci (eQTL) signals^24^ (Supplementary Table 2), revealed a total of 53 rare variants in 30 genes (underscored in Figure 2), distributed across 33 kindreds (33/50=66%).

Our previous analyses in Caucasian SLE enriched in childhood/juvenile-onset disease identified 10 SLE-associated genes validated by eQTL analysis present in over 5% of SLE probands, with *BLK, LYST, TYK2, UHRF1BP1* and *IKBKE* being the genes most commonly harboring rare variants^25^. In our Chinese cohort, *TYK2, C4A*, and *C4B* were the genes most frequently containing rare variants in Chinese cohort (Figure 3B). TYK2 promulgates signaling downstream of many cytokines including type I and type III interferons. None of the TYK2 variants, were predicted to be pathogenic by our in-silico prediction tools, however, these algorithms are often poor predictors of pathogenic gain-of-function variants, which compared with loss-of-function variants tend to interfere less with protein structures^26^.

#### De novo rare variants

De novo variants are known to explain two thirds of severe genetic disease^10^ and are a fertile ground for understanding disease pathogenesis and discovering novel drug targets. By comparing the sequencing data of the proband and their parents, we identified a total of 125 de novo variants in 93 genes amongst 33 probands.

Seventeen probands did not carry bona fide rare coding de novo variants. We found two probands (49 and 50) had an unusual number of *de novo* variants − 67 in a total in 42 genes (Figure 2); relatedness was confirmed genetically, as was the absence of variant reads in the parents of these two trios. By comparing the variant allele frequency (VAF) of de novo variants in the two families with an unusually high number of such variants to the remaining 48 families, we found a higher proportion of low VAF (10%-26%) de novo variants in these two families (Supplementary Figure 2A and Supplementary Table 4). This suggests a greater likelihood of somatic mutations − as opposed to gonadal parental mosaicism - in these cases^27^. While neither patient in family 49 and 50 has developed neoplastic disease to date, the possibility of undetected pre-neoplastic processes cannot be ruled out. Further investigation and long-term follow-up of these patients will be essential to clarify the clinical significance of these genetic findings.

We took advantage of the Gene4denovo WES database to analyze de novo rare variants in healthy control samples. Utilizing the same filtering criteria used for our cSLE cohort, we identified a total of 1619 de novo rare variants (including missense, nonsense, canonical splice site variants, and coding region indels), distributed in the coding regions of 1454 genes in 2049 healthy control individuals. The total number of de novo rare variants in our cSLE cohort was significantly higher compared to the control group. This difference remained statistically significant even after excluding two probands (Families 49 and 50) who carried an unusually high number of de novo variants (Supplementary Figure 2B and Supplementary Table 5). A comparison of per-sample counts of rare de novo variants between the SLE cohort and controls also suggested a significantly higher genetic burden in cSLE patients (Supplementary Figure 2C). These results must nevertheless be taken with caution given the use of different sequencing platforms in SLE vs healthy controls.

In addition to the aforementioned variants in *NRAS* and *P2RY8*, three genes carrying de novo variants - *GADD45A, TET2*, and *PHRF1* - have been previously associated with SLE. GADD45A plays a crucial role in DNA repair and cellular stress responses, and its dysregulation has been linked to autoimmunity, including lupus^28^. TET2, a key regulator of DNA demethylation, has been implicated in immune dysregulation and lupus pathogenesis^29^. *PHRF1* is a well-established SLE GWAS gene, and an E3 ubiquitin ligase involved in genome integrity^30^. Additionally, LILRA6 has been implicated in rheumatoid arthritis and multiple sclerosis, while MCM10 has been identified in the context of immunodeficiency disease, with or without congenital cardiomyopathy. Thus, collectively, 6 genes (12%) show direct ties to immune diseases. For genes without established direct links to immune diseases, 23 genes (46%) are relevant to immunity (Supplementary Figure 2D and Supplementary Table 6).

We have previously shown that except for complement genes, most genes causing monogenic SLE or associated to lupus in GWAS are expressed in B cells, and a significant fraction of them are most highly expressed in age-associated B cells (ABCs). To investigate the likelihood that the genes carrying de novo variants in our cohort are contributing to the SLE phenotype, we first investigated their expression pattern. Using the online transcriptome GENEVESTIGATOR database, we plotted immune cell-specific expression of genes with de novo variants. We were intrigued to see 50 genes carrying de novo variants were expressed in B cells. Amongst them, 19 genes − *ACTR5, AGO1, ASAP1, CTBP1, DHX8, FAM136A, GFM2, LRRC37B, NRAS, P2RY8, PDE12, PHRF1, PLAGL1, PNRC1, PPARGC1B, TET2, TNRC6B, WDR44* and *ZNF232* − were highly expressed in ABCs, and 12 genes − *ABLIM3, ATRNL1, DAND5, GPRC5C, LILRA6, OR10D3, PLEC, RASAL1, SLC35G5, SLC51B, TLX3* and *WNT6* − were highly expressed in plasma cells (Supplementary Figure 3A). Amongst the 42 genes carrying de novo variants in the two outlier probands, 26 showed expression in immune cells, with EPC1 and WASHC4 (Supplementary Figure 3B) being highly expressed in ABCs and STAB2 highly expressed in plasma cells. Highly expression in ABCs together with its role in DNA repair makes *EPC1* a possible SLE contributing gene. *WASHC4* encodes a component of the WASH complex, which functions in the intracellular transport of endosomes.

### Pathway enrichment analysis and visualization of rare variants

**Figure 4:**
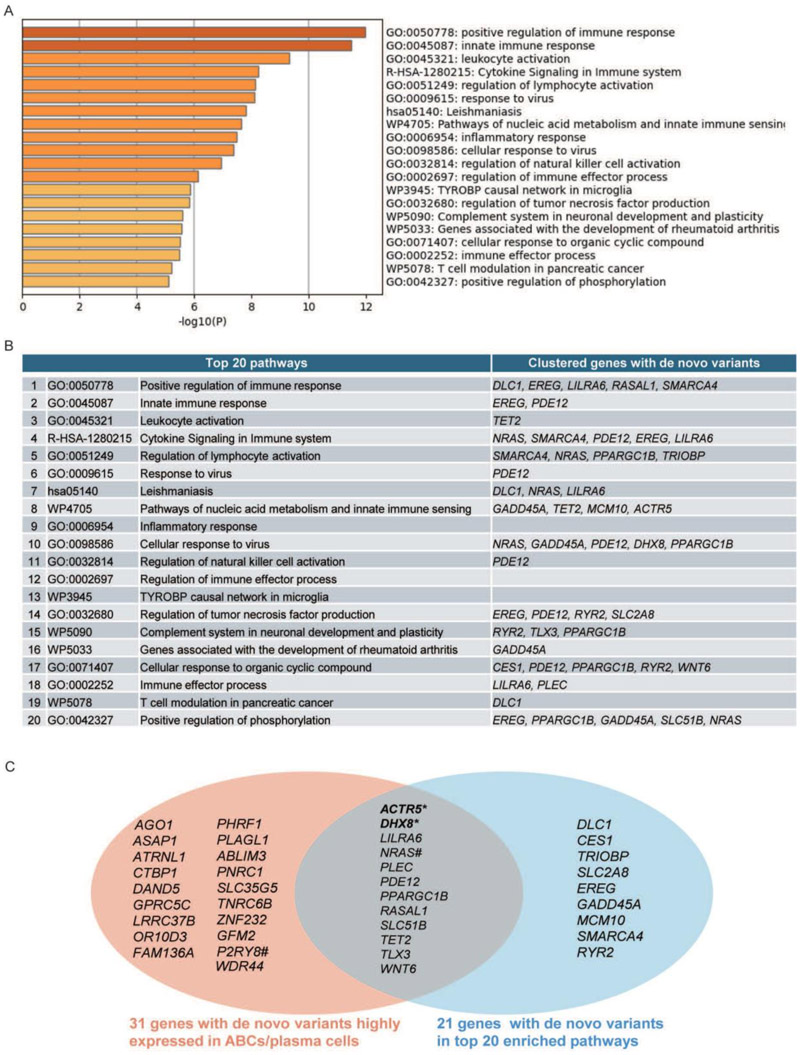
Variants based signaling pathway gene set for enrichment analysis. All selected genes with rare variants carried by patients were processed with online tool “Metascape” for enrichment analysis. Four pathway analyses were included: GO, KEGG, Reactome and Wiki. (A) The top 20 pathways were automatically generated with the -log10 p value>4 after the enrichment analysis. (B) List of genes of de novo variants that clustered in each of the top 20 pathways. (C) Venn diagram shows the overlap between the 31 genes carrying de novo variants highly expressed in ABCs /plasma cells and the 21 genes carrying de novo variants belonging to the top 20 enriched pathways. *: Taken further for functional validation genes, #: Genes with variants already reported to be pathogenic.

To obtain further insights as to potential contribution of de novo variants to SLE in the probands, we asked whether the genes harboring the variants belong to SLE-related pathways. For this, we conducted a pathway enrichment analysis using the online tool Metascape. A first analysis included Gene Ontology (GO) pathways, Kyoto Encyclopedia of Genes and Genomes (KEGG) pathways, Reactome pathways & Wiki pathways, including all the genes with rare variants identified in the probands, including SLE monogenic genes, SLE GWAS genes and the 50 genes with *de novo* variants. Two probands that had an unusual number of *de novo* variants − 42 in total − and were excluded from this analysis. Metascape generated the top 20 statistically significant pathways, with “positive regulation of immune response” emerging as the most significant pathway (Figure 4A, 4B). Other SLE-related pathways included innate immune response, immune cell activation, cytokine signaling, cellular responses to virus, nucleic acid metabolism, inflammatory response, regulation of immune effector process, and regulation of phosphorylation. Nearly half of the genes (21 out of 50) with de novo variants clustered among these top 20 pathways (Figure 4B), with some genes like *PDE12, EREG, NRAS, PPARGC1B, GADD45A*, and *LILRA6* appearing in 7, 5, 5, 5, 4, and 4 clusters respectively. Besides *DLC1* and *EREG, RASAL1* (RAS protein activator-like 1) also formed part of the top SLE pathway − “positive regulation of the immune response”. The novel *RASAL1* W203X variant is predicted to change the length of the protein. RASAL1 belongs to the GAP1 family of GTPase-activating proteins, acting as a suppressor of RAS function to control cellular proliferation and differentiation^31^. Considering its high expression in plasma cells, a loss-of-function variant in *RASAL1* would be good candidate to contribute to cSLE. Together these results suggest that a significant proportion of *de novo* variants in cSLE probands may contribute to disease pathogenesis and are worth investigating further.

### Functional validation of the promising de novo variants

To further probe whether some of the de novo variants occurring in genes not previously linked with SLE may contribute to disease, we selected two genes for functional analysis amongst the 12 genes that are both expressed most highly in ABCs/plasma cells and belong to the top 20 SLE pathways identified through enrichment analysis: *DHX8* and *ACTR5* (Figure 4C).

**Figure 5:**
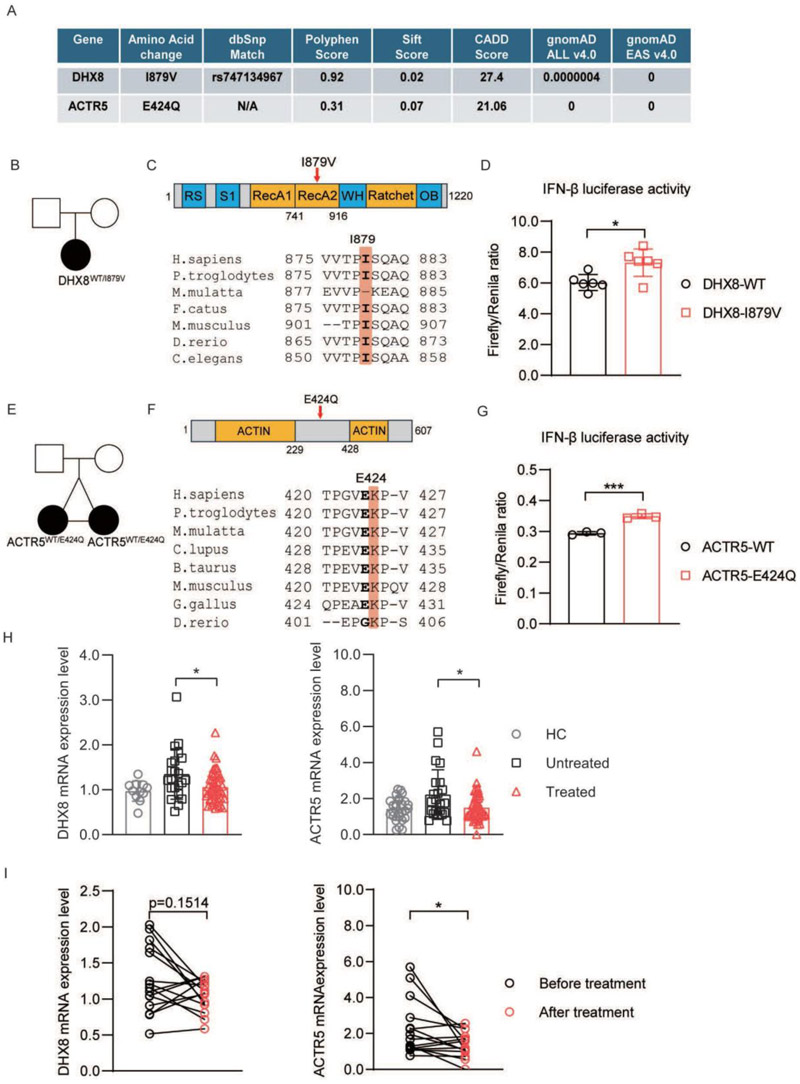
Characteristics of de novo rare variants in two SLE trios. (A) Bioinformatic predictions of protein damage for each de novo variant based on PolyPhen, SIFT, and CADD score. The gnomAD v4.0 allele frequency, and SNP database (dbSNP) match are shown. EAS = East Asian. (B)(E) Pedigrees of probands carrying de novo rare variants in DHX8 and ACTR5. (C)(F) Schematic representation of protein domain and evolutionary conservation of each residue. (D)(G) IFN-β luciferase assay with indicated wild-type and mutant cDNAs. (H) (I) Left: DHX8 mRNA expression in SLE patients’ PMBCs. HC: healthy control (n=11), untreated patients (n=22), treated patients (n=47), patients before and after treatment (n=15). Right: ACTR5 mRNA expression in SLE patients’ PMBCs. HC: healthy control (n=27), untreated patients (n=22), treated patients (n=47), patients before and after treatment (n=15). P values determined by unpaired t test (D, G), Mann-Whitney t test (H, I). *, P < 0.05; **, P < 0.01; ***, P < 0.001; Graphs depict mean with SD.

DHX8 is an ATP-dependent RNA helicase involved in pre-mRNA splicing^32^. It is required for the release of mature mRNA from the spliceosome^32^. The de novo variant I879V is located in the RecA2 domain, which forms the helicase core responsible for its helicase activity. This variant is evolutionarily conserved among species and predicted to be damaging by Polyphen, SIFT and CADD algorithms (Figure 5A-C). DHX8 mRNA expression levels were elevated in untreated SLE patients compared to those receiving treatment (Figure 5H, 5I). A parallel trend was observed in the expression of interferon-stimulated genes (ISGs) in these patients (Supplementary Figure 4). A luciferase assay upon transfection of WT or mutant protein into HEK293 cells demonstrated that the DHX8 I879V variant causes increased IFN-β activity compared to the wild type protein (Figure 5D), suggesting that the variant may positively regulate type I IFN pathway.

ACTR5, also known as an acting-related protein 5 (ARP5), is one of the subunits of the ATPase-dependent chromatin remodeling complex INO80, which is involved in repair of UV-damaged DNA and double-strand breaks in DNA^33^.This gene forms part of the “nucleic acid metabolism and innate immune sensing” pathway. Of note, two identical twins from proband 47 both carry the novel and de novo variant E424Q in *ACTR5*, and both twins exhibit a UV-sensitive clinical lupus phenotype. The variant is evolutionarily conserved and predicted to be deleterious by CADD (Figure 5E, 5F). A luciferase assay showed that E424Q in ACTR5 led to increased IFN-β activity (Figure 5G). Additionally, *ACTR5* mRNA was higher in untreated compared to treated SLE patients (Figure 5H, I). A similar trend was observed in interferon-stimulated gene (ISG) expression in these SLE patients (Supplementary Figure 4). Together these findings suggest that the function of ACTR5 in DNA repair may be important to maintain immune tolerance and prevent SLE, particularly upon UV-damage.

## Discussion

Unlike the common variants identified through GWAS, rare variants tend to have a larger effect size and are frequently found within protein-coding regions, offering insights into disease mechanisms that may be missed by traditional approaches. Notably, de novo rare variants, which arise spontaneously and are not either not inherited from parents, or inherited from mosaic parents, can be especially informative in understanding severe forms of sporadic cSLE. Trio-based genetic analysis provides valuable information into the pattern of inheritance of gene variants and uncovers the presence of de novo variants. The latter, when shown to be pathogenic, can offer profound insights into the mechanisms underpinning disease. Whilst some studies have investigated the burden of rare gene and de novo variants in cSLE, this is the first study in a Chinese cohort.

In our cohort, all probands carried at least one rare variant in a known SLE gene with some children carrying up to 10. We only call monogenic SLE in 2 probands (4%) with sufficient degree of confidence. This is within the 3.5-7% monogenic diagnostic range in published British and French cohorts^23, 34^. Our focus on Asian populations is particularly important given the known ethnic variations in SLE prevalence and severity. For example, Asian populations generally show higher SLE prevalence and more severe disease manifestations compared to European populations^35^.

In our analysis, *TYK2* emerged among the top 5 genes in both Chinese and Caucasian cohorts^25^. TYK2 binds to the type I IFN receptor complex and gain-of-function variants in TYK2 enhance cytokine signalling^36^. These findings carry significant therapeutic implications, as selective TYK2 inhibitors could mitigate IFN-driven responses in SLE^37^. Current clinical trials, including those of deucravacitinib, have shown promising results in SLE treatment^38^. The cross-population identification of rare TYK2 variants highlights its crucial role in SLE pathogenesis, paving the way for more precise genetic testing, personalized therapies, and novel targeted treatments.

In this study, rare and coding de novo variants were identified in all probands from 50 trios. The median number of de novo coding variants was 1, which coincides with reports in the literature^39^, with a range of 0-16 variants per proband. In the context of cSLE, de novo variants are especially informative because they can explain more severe forms of the disease. Since these mutations are not inherited (or they are inherited from parents with gonadal mosaicism), they frequently escape evolutionary filtering, resulting in more damaging effects on essential biological processes.

Consequently, pathogenic de novo variants in cSLE are often found in genes involved in immune regulation, DNA repair, and other critical cellular functions. Disruptions in these pathways can lead to an overactive immune response, increasing the risk of severe manifestations. Thus, studying de novo variants in cSLE can uncover novel genes and pathways essential to disease development, paving the way for targeted therapeutic interventions. Notably, in the past two years, three different reports have described de novo variants in TLR7 that confer gain-of-function, in children with early onset lupus-like disease^3, 40, 41^.

We took a combination of two approaches to prioritize the de novo variants: 1) being amongst the top 20 SLE GO-pathways (21/50=42%) and 2) expression in ABC/plasma B cells (31/50=62%). This identified 12 genes (12/50=24%) that fulfilled both criteria, and thus constitute promising candidate lupus genes. Notably, this group contained a gene previously implicated in the causation of SLE (*NRAS*)^17–20^. Initial functional validation of ACTR5 and DHX8 showed that both enhanced type I IFN signaling. Increased type I IFN activity is a hallmark of SLE, promoting immune activation and autoantibody production^42^. Monogenic forms of SLE often involve mutations in genes that modulate the metabolism of nucleic acids (e.g.,*DNASE1, DNASE1L3, IFIH1, RNASEH2A/B/C, ADA2*, and *SAMHD1*) or mediate nucleic acid sensing (e.g., *TLR7, UNC93B1, IFIH1, ACP5, TMEM173*), leading to excessive type I IFN signaling^43^. Type I IFN reporter assays effectively prioritize variants for further investigation. Once promising variants are identified through functional screening, definitive evidence of SLE causation may be obtained by assessing the effects of these variants in primary immune cells and animal models of lupus. Our multi-faceted approach, combining bioinformatic predictions and functional assays, offers a strategy to identify and prioritize de novo variants that may contribute to SLE pathogenesis and uncover potential therapeutic targets.

It was interesting that more than half of de novo variants occurred in genes that were not previously associated with SLE or autoimmunity but were highly expressed in age-associated B cells (ABCs), a B cell subpopulation thought to be pathogenic in SLE^44^. ABCs are known to be enriched in SLE patients and exhibit a strong correlation with autoantibodies and disease activity scores. ABCs have been shown to efficiently differentiate into PCs, and their absence is associated with a reduction in autoantibodies and histologic manifestations of disease^45, 46^. The detailed mechanisms governing ABC function and their complex interactions with other immune cells are currently the focus of intensive investigation^13, 47^.

Our study focused on rare coding variants associated with SLE, employing a MAF threshold of <0.005 for variant filtration. While this cut-off value allowed us to concentrate on rare variants, we acknowledge that the choice of 0.005 is somewhat arbitrary and may exclude potentially relevant variants above this threshold. It has been very well established that non-coding and more common variants identified by GWAS can also exert functional effects and act in a polygenic manner to confer SLE risk and larger studies using WGS will be needed to investigate the contribution of rare and common alleles in coding and non-coding regions combined, ideally including polymorphisms in the HLA region. Some of the novel putative SLE candidate genes and variants reported here may help further our understanding of SLE pathogenesis relevant to Asian populations and once validated, harnessed for development of personalized therapies. Although our sample size of 50 trios may seem limited for a complex disease, our findings represent an important first step toward elucidating the intricate genetic contributions to SLE. Future large-scale, collaborative studies across diverse populations will be essential to validate and extend these findings, ultimately refining our understanding of SLE pathogenesis.

## Supplementary Material

Supplementary Table 1

Supplementary Table 2

Supplementary Table 3

Supplementary Table 4

Supplementary Table 5

Supplementary Table 6

Supplementary Figure

Supplementary Figure 1

Supplementary Figure 2

Supplementary Figure 3

Supplementary Figure 4

## Figures and Tables

**Table 1:** Clinical features of cSLE patients

Diagnosis	SLE
Sex, female	78.4% (40/51)
Age at onset (yr), mean ± SD	10.8 ± 3.46
SLEDAI-2k, mean ± SD	9.96 ± 4.91
Alopecia	25.4% (13/51)
Arthritis	35.3% (18/51)
Serositis	3.9% (2/51)
Leukopenia	19.6% (10/50)
Lymphopenia	7.8% (4/51)
Renal	39.2% (20/51)
ANA	100% (24/24)
Anti-dsDNA	69% (29/42)
Anti-SM	9.8% (5/51)
Anti-RNP	17.6% (9/51)
Anti-SSA	27.5% (14/51)
Anti-SSB	13.7% (7/51)
Cardiolipin antibodies	6.1% (3/49)
β-2 glycoprotein I antibodies	0% (0/45)
Consumed C3	52.9% (27/51)
Consumed C4	37.2% (19/51)
Seizures	0% (0/51)
Psychosis	0% (0/51)
Myelitis	0% (0/51)

Data were presented in the form of % (n/total). RNP, ribonucleoprotein; ANA≥1:80 as positive; anti-dsDNA≥15 IU/ml as positive; C3, complement C3; C4, complement C4;
