# Supplementary material for: Trio whole exome sequencing in Chinese childhood-onset lupus reveals novel candidate genes
Source: Arthritis Rheumatol. Author manuscript; Available in PMC 2025 Nov 1. (PMC7617808; doi:10.1002/art.43243)
Supplement: Supplementary Figure [file EMS206315-supplement-Supplementary_Figure.docx]

## SupplementaryFigure1Deleteriousnessanddistributionofrarevariantsin the SLE cohort.

A) Deleteriousness of de novo rare variants (left) and rare variants in SLE-associated genes (right) based on PolyPhen-2, SIFT, and CADD scores. Likely tolerated: predicted benign/likely benign by at least two tools; Likely damaging: predicted pathogenic/likely pathogenic by two at least two tools; Uncertain:conflictingpredictionsacrosstools (somepredictiontoolsdidnotprovideascore).Detailed information is in SupplementaryTable 2. (B) Median numbers of rare variants in monogenic SLE genes,SLEGWASgenes,andgenes withdenovorarevariantsintwoagegroups(0–9yearsvs.10– 15 years).

## SupplementaryFigure2ComparisonofdenovorarevariantsinSLEpatients and healthy controls.

(A) Variant allele frequency (VAF) distribution in de novo variants in families 49 and 50 compared to theremainingfamilies.ThevariantsaregroupedintothreeVAFcategories:VAF>26%(orange),VAF

=16%-26%(purple),andVAF=10%-16%(blue).(B)TotalnumberofdenovorarevariantsinSLE patients versushealthycontrols(Gene4DenovoWESdatabase).Pvalues weredeterminedbythe Chi-Square test. (C) Per-sample counts of de novo rare variants in SLE probands and controls. P values were determined bythe Mann-Whitneyt test (*, P<0.05). (D)Association of genes with de novo variants to diseases. Detailed information is provided in Supplementary Table 6.

## SupplementaryFigure3Cell-specificexpressionofgeneswithrarevariants identified in SLE patients.

Heatmapsillustratethelog2-transformedcell-specific expressionofselectedgeneswithraredenovo variants in 31 probands (A), and other two probands (49 and 50) (B), monogenic genes with variants (C), or SLE GWAS genes with variants (D) identified in 50 SLE trios. Heatmaps have been created usingtheonlinetranscriptomedatabaseGENEVESTIGATOR(AffymetrixHumanGenomeU133Plus 2.0 array) and a compendium-wide analysis using conditional search of cell types. Hierarchical clustering is performed by cell type. PMN, polymorphonuclear neutrophil; NK T cell, natural killer T cell;ABC, aged-associated B cell. MUC21, OR6C65, ZNF98 and CNOT2 were not included in the heatmap, since they were not available in the GENEVESTIGATOR database.

## SupplementaryFigure4Interferon-stimulatedgenes(ISG)mRNAexpression from SLE patients’PMBCs before & after treatment.

(A) OAS1 mRNAexpression level. (B) MX1 mRNAexpression level. (C) IFIT1 mRNAexpression level. HC: healthy control (n=13), untreated: SLE patients before treatment (n=22), treated: SLE patientsaftertreatment(n=47).PvaluesdeterminedbyMann-Whitneyttest. *,P<0.05;**,P<0.01;

***,P<0.001;GraphsdepictmeanwithSD.

**SupplementaryTable 1 Detailed clinical information of SLE patients Supplementary Table 2 List of SLE-associated genes Supplementary Table 3 Primer sequences used for Realtime-PCR SupplementaryTable4Rarevariantsidentifiedin50cSLEprobands**

**SupplementaryTable5Denovorarevariantsidentifiedinhealthycontrols (gene4denovo WES database)**

**SupplementaryTable6Overviewoftherelationshipbetweengeneswithde novo variants and disease**
