# Supplementary figures and images for: Trio whole exome sequencing in Chinese childhood-onset lupus reveals novel candidate genes
Source: Arthritis Rheumatol. Author manuscript; Available in PMC 2025 Nov 1. (PMC7617808; doi:10.1002/art.43243)

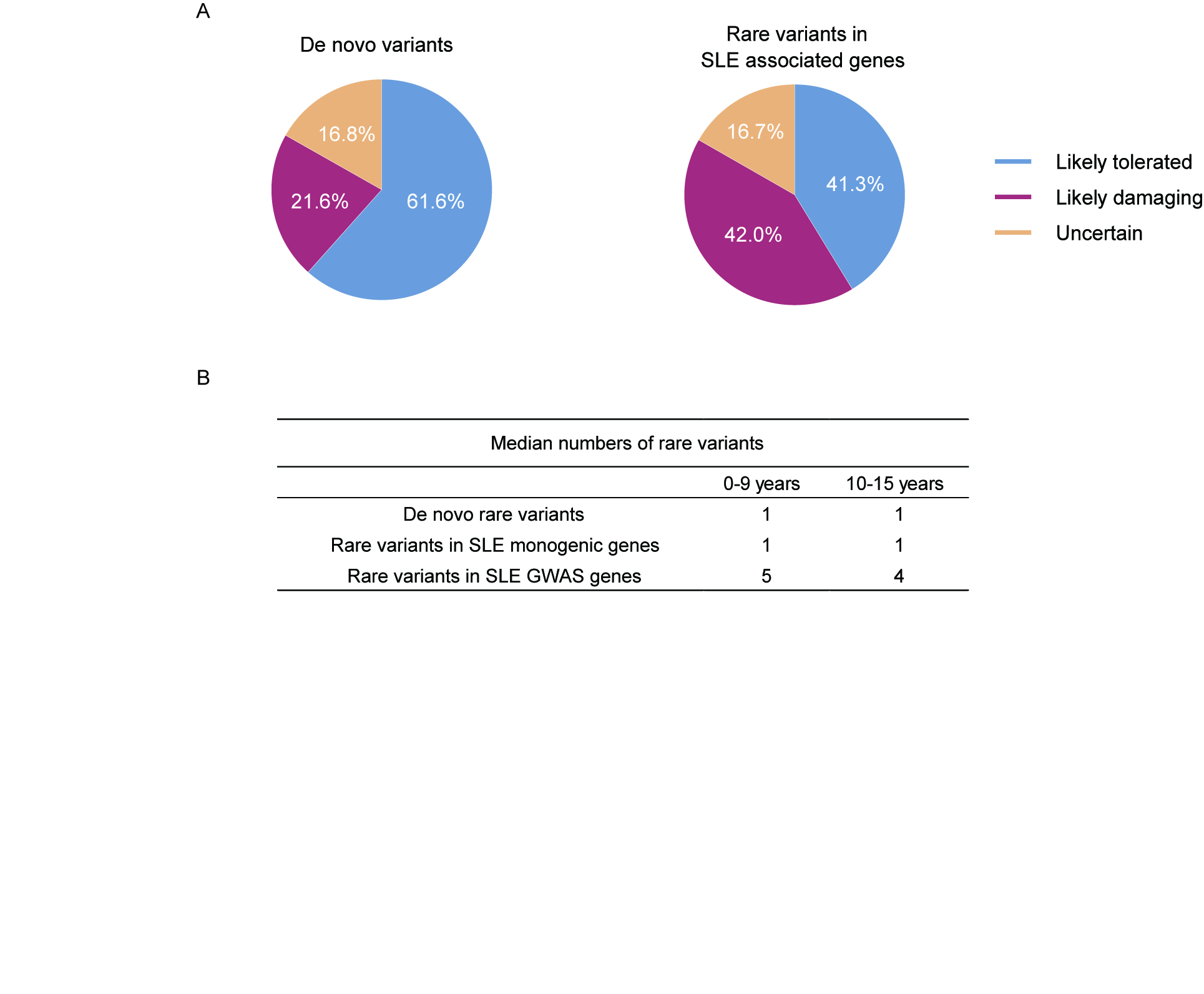

Supplement: Supplementary Figure 1 [file EMS206315-supplement-Supplementary_Figure_1.tif]

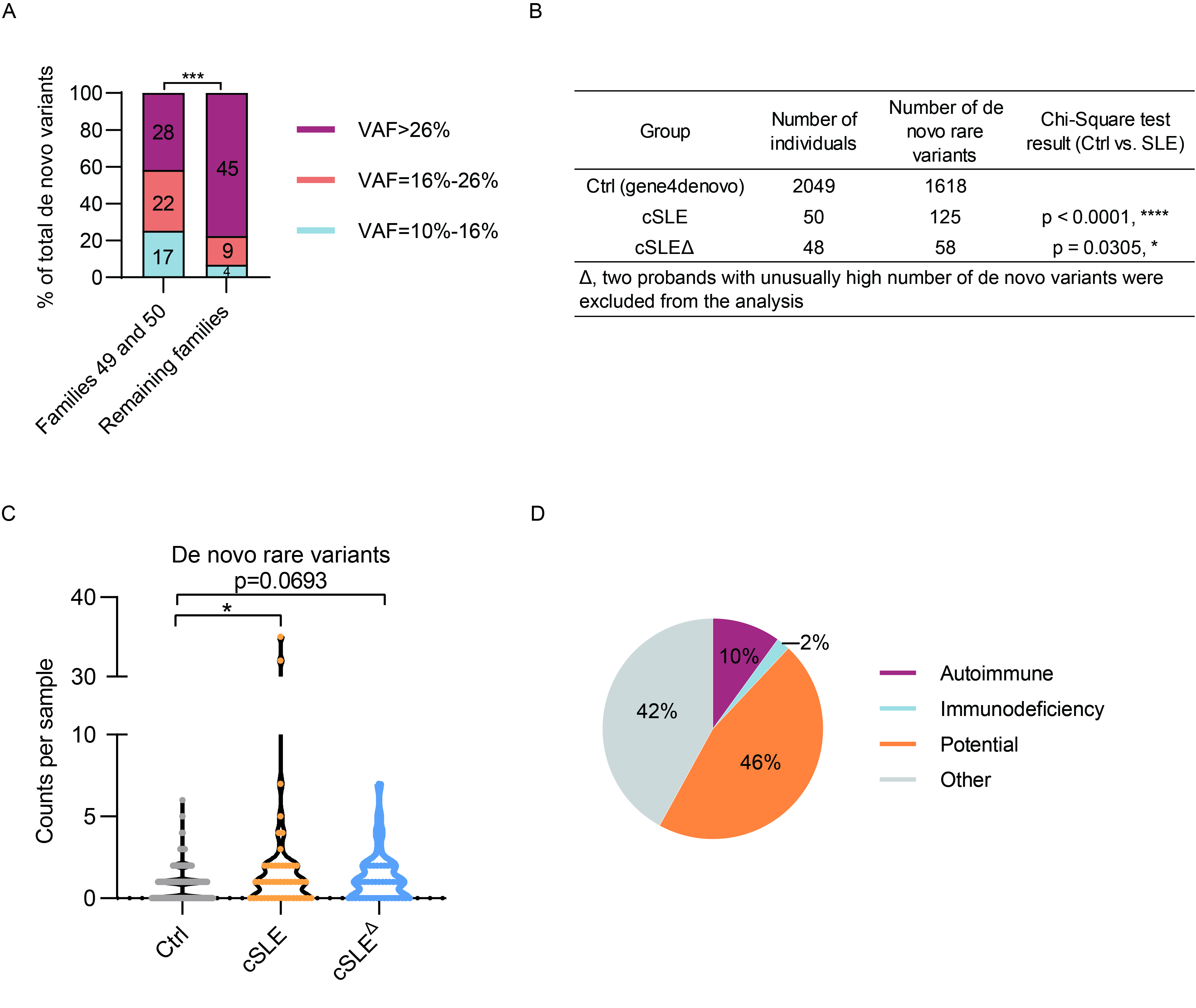

Supplement: Supplementary Figure 2 [file EMS206315-supplement-Supplementary_Figure_2.tif]

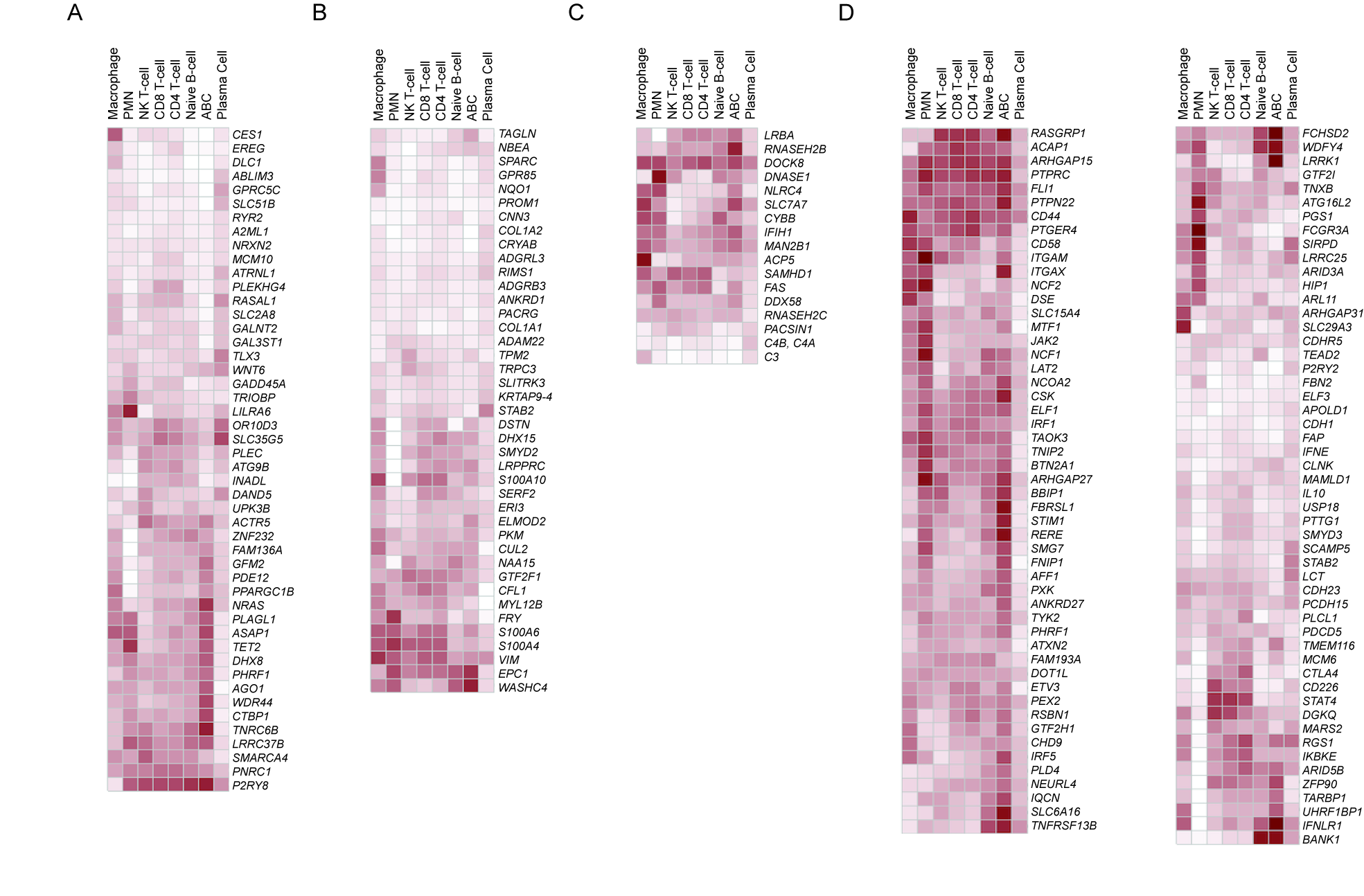

Supplement: Supplementary Figure 3 [file EMS206315-supplement-Supplementary_Figure_3.tif]

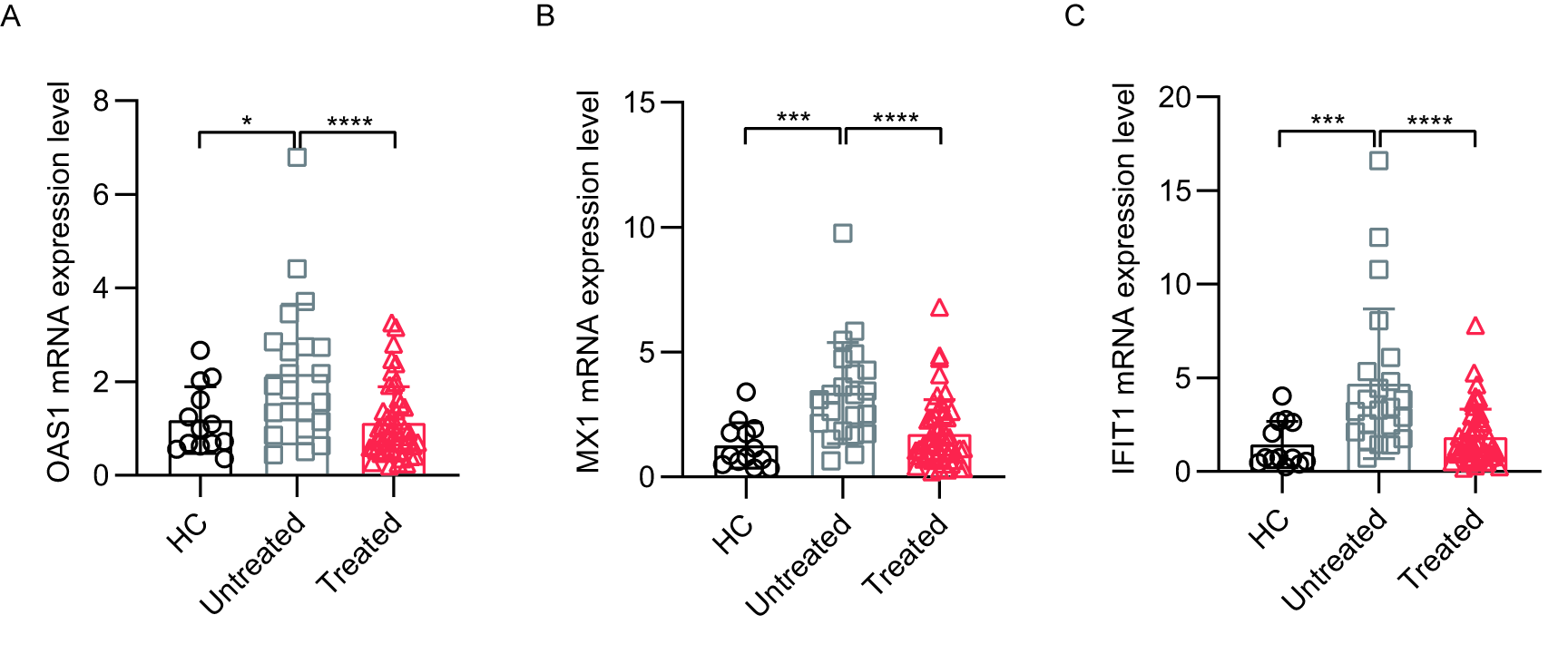

Supplement: Supplementary Figure 4 [file EMS206315-supplement-Supplementary_Figure_4.tif]
